# Caregiver burden in parents of children with neurological impairement and its relation with depression

**DOI:** 10.1192/j.eurpsy.2023.1750

**Published:** 2023-07-19

**Authors:** A. Mellouli, S. Zouari, N. Smaoui, W. Bouchaala, I. Gassara, O. Jallouli, R. Feki, S. Ben Ncir, F. Kamoun, M. Maâlej, C. Charfi Triki

**Affiliations:** 1 Psychiatry C Department; 2 Child Neurology Department, Hedi Chaker University Hospital; 3 LR19ES15 laboratory of Child Neurology, Sfax medical school-Sfax university, Sfax, Tunisia

## Abstract

**Introduction:**

Caregiving negatively affects the psychological and physical health of the caregivers, especially in parents of children with neurological impairement (NI).Furthermore, the behavior and demands of the patient make the caregivers encounter increased stress levels and negative thoughtsabout the future that may lead to depression in caregivers.

**Objectives:**

To assess the relationship between caregiver burden and symptoms of depression in parents of children with NI.

**Methods:**

A total of 33 caregivers of children with NI, participated in this cross-sectional, descriptive and analytical study, carried out in Child Neurology Department of the University Hospital in Sfax (Tunisia), between February and April 2021.

The Zarit-Caregiver-Burden-Scale (Zarit-CBS) and the Beck Depression Scale were administered.

**Results:**

The average age of the caregivers (27 mothers and 6 fathers) was 38,33 years ± 6,53 years. Among the parents, 81,81% didn’t exceed the secondary educational level and75,75% of them had an irregular occupation.

The average age of the children (21 boys and 12 girls) was 7,58±4,29 years.Near to the half of them (51,51%) had intellectual disability.Over 54.54% of the children had a functional independence, while 21.21% required help in walking and 24.24% were unable to walk.

The intervention was based on motor rehabilitation (57,57%), adequate equipment (24,24%), ergotherapy (45,45%) and speech therapy (60,6%).After the intervention, 63,63% of children had an improvement and 30,3% had a stationary state.

The mean score of Zarit-CBS was 52,45±14,26. The caregiver burden was noted in 96,96%.

The mean score of Beck was 9,33±5,48. The depression was noted in 78,78%.

The total Zarit-CBS score had positive correlation with Beck scores (p=0.038).

**Conclusions:**

There is a positive relationship between the caregiver burden and depression symptoms. Thus, effort should be made to relieve caregiver burden in parents of children with NI.

**Disclosure of Interest:**

None Declared

